# The Traditional Medicinal Plants* Cuphea calophylla*,* Tibouchina kingii*, and* Pseudelephantopus spiralis* Attenuate Inflammatory and Oxidative Mediators

**DOI:** 10.1155/2018/1953726

**Published:** 2018-04-24

**Authors:** Ana María Ramírez-Atehortúa, Lorena Morales-Agudelo, Edison Osorio, Oscar J. Lara-Guzmán

**Affiliations:** Grupo de Investigación en Sustancias Bioactivas, Facultad de Ciencias Farmacéuticas y Alimentarias, Universidad de Antioquia, Calle 70 No. 52-21, Medellín, Colombia

## Abstract

Aerial parts of* Cuphea calophylla, Tibouchina kingii*, and* Pseudelephantopus spiralis* have been used in Colombian traditional medicine for inflammation. However, the underlying mechanisms that could explain the anti-inflammatory actions remain unknown. This study aimed to elucidate the anti-inflammatory and cytoprotective effects of hydroalcoholic extracts from* C. calophylla *(HECC),* T. kingii *(HETK), and* P. spiralis *(HEPS) in LPS-stimulated THP-1 macrophages. Reactive oxygen species (ROS), nitric oxide (NO), and malondialdehyde (MDA) were monitored as inflammatory and oxidative markers. The inhibition of lipoxygenase (LOX) and cyclooxygenase (COX) activities in a cell-free system were also investigated. Antioxidant activities were determined using standard* in vitro* methods. All extracts inhibited the NO, ROS, and MDA levels. HETK showed the highest ROS production inhibition and the highest antioxidant values, whereas HETK and HEPS significantly decreased the cytotoxicity mediated by LPS. The release of MDA was reduced significantly by all extracts. Moreover, the catalytic activity of LOX was inhibited by HECC and HETK. HECC was a more potent reducer of COX-2 activity. All extracts effectively suppressed COX-1 activity. In summary, these results suggest that HECC, HEPS, and HETK possess anti-inflammatory properties. Therefore, these plants could provide a valuable source of natural bioactive compounds for the treatment of inflammatory-related diseases.

## 1. Introduction

Inflammation is one of the first physiological responses of the body to a chemical, mechanical, or biological injury induced in the tissue; it involves activation of the immune system to act as a defensive barrier [1, 2]. During inflammation, macrophages constitute a principal component and play a crucial role in the initiation, maintenance, and resolution of this process owing to their immunomodulatory, phagocytic, and antigen presentation functions [3]. Once the macrophage contacts an activating stimulus, for example, lipopolysaccharide (LPS), a component of the outer membrane of Gram-negative bacteria and belonging to the so-called pathogen-associated molecular patterns (PAMPs), is recognized by toll-like receptor-4 (TLR4), which is part of a family of receptors that recognize patterns (PRRs) and express these cells. TLR4 activates multiple intercellular signaling pathways, including MyD88-dependent and MyD88-independent, mitogen-activated protein kinase (MAPK) families and others, which lead to the release of a wide variety of inflammatory mediators for host defense, such as tumor necrosis factor-*α* (TNF-*α*), interleukins (IL-6, IL-1*β*), reactive oxygen, and nitrogen species (ROS/RNS), as well as eicosanoid derivatives (prostaglandins and leukotrienes) by activation of the catalytic activity of the enzymes lipoxygenase (LOX) and cyclooxygenase (COX) [2, 3]. High-dose LPS induces a robust yet transient inflammatory response, being an acute response of rapid onset and of generally short duration, whereas a “subclinical” low dose causes low grade yet persistent inflammatory responses from the host, as reflected in mildly sustained levels of inflammatory mediators [4]. In fact, overproduction of inflammatory mediators from LPS-activated macrophages causes deleterious damage and produces more inflammation, and the persistent inflammation increases the development of chronic and degenerative diseases [3, 5]. Several investigations have demonstrated the contribution of exacerbated activation of macrophages in the pathogenesis of diverse chronic diseases such as cardiovascular disease, neurodegenerative disorders, autoimmune diseases, sepsis-related multiorgan failure, and tumorigenesis [6]. In these pathological processes, a wide range of biologically active molecules that participate in both beneficial and detrimental outcomes in inflammation are produced. Consequently, therapeutic interventions targeting macrophages and their products open new approaches for controlling inflammatory diseases [3].

In recent years, there has been a growing interest in the ingredients and extracts used in traditional medicine systems, which stand out as potential alternatives for the treatment of the inflammatory process [7, 8]. In spite of the wide number and variety of anti-inflammatory drugs used to control symptoms and to prevent further development of illnesses, conventional drugs present some side-effects that cause damage when consumed over long periods of time and increase the costs in healthcare [9]. Despite the fact that a plant-based medicinal product does not mean that it is nontoxic, several medicinal plants have been screened with the aim of developing alternative drugs with increased potency and fewer adverse effects than existing drugs [10] and are recognized by their large number of bioactive secondary metabolites with the ability to affect multiple targets of signaling pathways and thus have several mechanisms to mitigate inflammation [11]. Therefore, to take advantage of traditional knowledge, multiple studies are currently being carried out to evaluate the anti-inflammatory potential of ingredients and extracts on cellular models using mainly human and murine macrophages as well as key enzymes in the inflammatory process like LOX and COX [12, 13].

In Colombia, the use of medicinal plants for treatment and prevention of diseases is a common practice in rural areas [14]. Eastern Antioquia is a region of special interest due to its diversity and long tradition of the use of wild and cultivated species for the treatment of symptoms associated with inflammatory processes [15]. Because of the close relationship between oxidative processes mediated by free radicals and inflammation [16], the antioxidant profile of 11 plants with high traditional anti-inflammatory use was evaluated in a ethnopharmacological study carried out in this region [17].* Cuphea calophylla *Cham. & Schltdl. (Lythraceae),* Tibouchina kingii *Wurdack (Melastomataceae), and* Pseudelephantopus spiralis *(Less.) Cronquist (Asteraceae) were those with better activity. Traditionally, the aerial parts of these plants with local names of Yerba buenilla, Lengua de vaca, and Coquito, respectively, are extensively used in folk medicine for the cure of many disease conditions related to inflammatory processes, both acute and chronic [15, 17]. In consideration of the activity of these species on the inflammatory process not having yet been reported, they were selected for anti-inflammatory activity evaluation in a cellular model using human THP-1 macrophages. Thus, to contribute to the preservation of traditional knowledge and confirm this medicinal use, the anti-inflammatory activity from each extract was investigated in LPS-activated macrophages to assess its cytoprotective effect, modulation of ROS, NO production, and malondialdehyde (MDA) reduction as a lipid peroxidation marker of oxidative/nitrosative stress [18, 19]. Finally, the inhibitory capacity of key inflammatory enzymes related to eicosanoid production such as COX-1, COX-2, and 15-LOX [20, 21] was also evaluated in a cell-free system.

## 2. Material and Methods

### 2.1. Chemicals and Reagents

2,4,6-Tris (2-pyridyl)-s-triazine (TPTZ), 2,2-azobis (2-methylpropionamidine) dihydrochloride (AAPH), Folin Ciocalteau reagent 2N, gallic acid, (±)-6-hydroxy-2,5,7,8-tetramethylchromane-2-carboxylic acid (Trolox), fluorescein sodium salt, 2-thiobarbituric acid (TBA), 1,1,3,3-tetramethoxypropane (TEP; MDA standard), dimethyl sulfoxide (DMSO), phorbol 12-myristate 13-acetate (PMA), lipopolysaccharide (LPS), and Triton X-100 were obtained from Sigma-Aldrich Co. (Saint Louis, Missouri, United States). Butylated hydroxytoluene (BHT), FeCl_3_, sodium carbonate, and HPLC grade solvents including methanol, acetonitrile, and hydrochloric acid (HCl) were obtained from Merck KGaA (Darmstadt, Hesse, Germany). Na_2_HPO_4_ and KH_2_PO_4_ were obtained from Carlo Erba reagents (Sabadell, Barcelona, Spain). Sodium acetate and trichloroacetic acid were obtained from Avantor J.T. Baker® (Upper Saucon, Pennsylvania, United States). Acetic acid was obtained from Mallinckrodt Pharmaceuticals (Chesterfield, Derbyshire, United Kingdom). Trypan blue was from Alfa Aesar (Haverhill, Massachusetts, United States). Phosphate buffered saline (PBS), fetal bovine serum (FBS), and RPMI cell culture media were from Gibco™ ThermoFisher Scientific (Waltham, Massachusetts, United States). Tris base UltraPure was from Amresco (Solon, Ohio, United States).

### 2.2. Plant Material

*C. calophylla, T. kingii*, and* P. spiralis *were collected in Guarne and Santuario, municipalities of East Antioquia, and the identification was made by comparison with specimens from the collection of the University of Antioquia's Herbarium (HUA). The vouchers are H. J. Sarrazola 890, 889, and 892, respectively. Fresh material was washed with distilled water and dried at 40°C for 5 days in a drying oven. All dried samples were mechanically blended until a homogeneous particle size was obtained to finally be stored at room temperature protected from light and moisture.

### 2.3. Extract Selection

Extract selection was made through chromatographic and antioxidant screening approaches. First, from each plant, four extracts were prepared with 100 mg of vegetal material in 1 mL of ethanol-water (70 : 30 v/v), ethanol, methanol, or water in a temperature-controlled sonication bath (Elma P60H, Singer, Germany) at 30 ± 5°C for 50 min. Then, the extracts were centrifuged at 161 rcf for 20 min to collect the supernatants. Chromatographic fingerprints were determined using a High-Resolution Liquid Chromatograph coupled to a Diode Array Detector (HPLC-DAD) Agilent series 1200 (Agilent Technologies; Palo Alto, CA, United States) equipped with vacuum degasser, autosampler, and quaternary pump. The peaks' separation and the fingerprints were performed on a reverse phase Agilent Zorbax SB RRHT (StableBond, Rapid Resolution High Throughput®) C18, 50 mm × 4.6 mm, 1.8 *μ*m, operating at 35°C and a flow rate of 1.0 mL/min. The mobile phase consisted of 0.1% (v/v) acetic acid in water (solvent A) and acetonitrile (solvent B) and it was developed with the following solvent gradient: 0–5 min, 5% B; 5–35 min 5–25% B; 35–55 min, 25–55% B. A volume of 5 *μ*L was injected and the chromatograms were monitored at 280, 320, 335, and 350 nm. The spectra were acquired 200–400 nm. Antioxidant activity was determined using the ferric reducing antioxidant power (FRAP) method, and it was adapted from a previously described procedure [17]. Chromatographic fingerprints and antioxidant activity for each extract of* C. calophylla, T. kingii*, and* P. spiralis *were compared and used as selection criteria for the best extraction solvent.

### 2.4. Hydroalcoholic Extract Preparation

Five g of vegetal material was extracted with 35 mL ethanol-water (70 : 30 v/v) in a temperature-controlled sonication bath according to the conditions mentioned above. The extracts were centrifuged for 20 min and the supernatants were recovered. A secondary extraction of the pellet with 25 mL ethanol-water was performed, and the supernatants were collected, combined, and dried using a Centrivap Cold Trap Labconco (Kansas City, MO, United States) to prepare stock solutions of each extract in 100% DMSO. Prior to use, working solutions in endotoxin-free PBS were prepared to evaluate the extracts at the final concentrations reported in the assays and to ensure a final DMSO concentration of less than 0.2%.

### 2.5. TPC and Antioxidant Activity

The total phenolic content (TPC) by Folin-Ciocalteu method, FRAP, and oxygen radical absorbance capacity (ORAC) value in the hydroalcoholic extracts were adapted from previously described procedures [17].

### 2.6. Anti-Inflammatory Activity

#### 2.6.1. Cell Culture

The human monocyte leukemia cell line THP-1 was obtained from American Type Culture Collection (ATCC, Rockville, Maryland, United States). THP-1 cells were cultured in RPMI 1640 medium supplemented with 10% FBS, 100 U/mL penicillin, and 100 *μ*g/mL streptomycin and maintained at 37°C in humidified atmosphere with 5% CO_2_. For experiments, THP-1 differentiation to macrophages was induced with 100 nM phorbol 12-myristate-13-acetate (PMA) for 3 days. Nonadherent cells were removed by aspiration of the supernatant followed by two washes with PBS and replacement with fresh medium without PMA at least 24 h before the experimental procedure.

#### 2.6.2. Cell Viability

In a 96-well plate, THP-1 macrophages (5 × 10^4^ cells) were seeded and treated with each of the extracts at different concentrations (1, 10, 50, and 100 *μ*g/mL) or 0.2% DMSO (vehicle control) for 48 h. Cell viability was tested by measuring the release of lactate dehydrogenase (LDH) in the medium using a Cytotoxicity LDH Assay Kit (Roche, Indianapolis, IN) per the manufacturer's protocol. The absorbance was measured at 490 nm using a UV/VIS PowerWave TM XS2 spectrophotometer (BioTek Instruments, Inc., United States). Total LDH for the positive control was obtained by exposing the cells to 2% Triton X-100 at 37°C for 30 min. The results are expressed as percentage cell viability.

#### 2.6.3. Cytoprotective Effect of the Extracts on LPS-Stimulated Macrophages

Cytoprotective assay was carried out according to a method previously described [22] with modifications. In a 24-well plate, 5 × 10^5^ THP-1 macrophages were incubated with each one of extracts at 10 *μ*g/mL or 0.025% DMSO (vehicle control) for 12 h. After treatment, the cells were stimulated by adding 10 ng/mL LPS (final concentration) for 12 h. A volume of 250 *μ*L of supernatant was collected to evaluate the cytoprotective effect by measuring the release of LDH and carrying out Nitrate/Nitrite assay. In addition, adherent cells and remnant of supernatant were removed to a freezer at –80°C for quantitation of MDA by TBARS assay.

#### 2.6.4. NO Production

Nitric oxide released from macrophages to supernatants was assessed by the determination of nitrite concentration in culture supernatant using Cayman's Nitrate/Nitrite Colorimetric Assay Kit (Cayman Chemical Co., Ann Arbor, MI, USA). Nitrates were converted to nitrites by nitrate reductase, and total accumulated nitrites were converted to an azo compound using Griess reagent. The absorbance was read at 540 nm, and the values were expressed as *μ*M of nitrites.

#### 2.6.5. TBARS

TBARS was carried out using the colorimetric technique described previously [23] with some modifications. Macrophages and supernatant remnants were suspended and lysed with SDS (1% final concentration). Then, 250 *μ*L of a solution of 0.67% TBA, 15% trichloroacetic acid, and 0.1 M HCl was added to 250 *μ*L of cell lysate and heated to 85°C for 30 min. After cooling in ice, the extent of lipid peroxidation was determined by the TBARS method, and the values were expressed as pmol of MDA, using TEP as standard. The absorbance at 532 nm was interpolated on the MDA calibration curve in a concentration range from 0.3125 to 20 *μ*M.

#### 2.6.6. ROS Production

In a 96-well black plate, 2 × 10^4^ THP-1 macrophages suspended in SBF-PBS (10 : 90 v/v) were incubated with each extract at 10 *μ*g/mL or 0.025% DMSO (vehicle control) at 37°C and 5% CO_2_ for 30 min. Then, final concentrations of 10 *μ*M DCFH-DA and 10 ng/mL LPS were added to each well. The intracellular ROS was measured at excitation and emission wavelengths of 485 and 520 nm, respectively.

#### 2.6.7. Lipoxygenase (LOX) Inhibition Assay

Soybean 15-LOX inhibitory activity was determined spectrophotometrically by using a Lipoxygenase Inhibitor Screening Assay Kit (Cayman Chemical Co., Ann Arbor, MI, United States), according to manufacturer's specifications, and arachidonic acid was used as substratum. The assay was carried out with extracts at 50 *μ*g/mL. The absorbance was measured at 490 nm, and the results were expressed as inhibition percentage of 15-LOX enzyme.

#### 2.6.8. Cyclooxygenase (COX) Inhibition Assay

Inhibitory activity on ovine COX-1 and human COX-2 was measured by using a COX Inhibitor Screening Assay Kit (Cayman Chemical Co., Ann Arbor, MI, United States) following the manufacturer's instructions based on measuring prostaglandin (PG) by ELISA. The inhibitory assay was developed with 50 *μ*g/mL extracts, and Indomethacin (0.02 *μ*M) was used as a positive control. The absorbance was measured at 420 nm. The effect of the distinct species on proinflammatory mediators was evaluated by calculating the inhibition percentage of PGF_2*α*_ production.

### 2.7. Statistical Analysis

The results were expressed as the mean ± SD. All statistical analyses were performed using GraphPad Prism 5 (GraphPad Software Inc., San Diego, CA, USA). One-way analysis of variance and Dunnett and Bonferroni multiple comparison test were performed to evaluate significant (*p* < 0.05) differences between samples and controls and between samples, respectively.

## 3. Results and Discussion

### 3.1. Extract Selection

**Figure 1:**
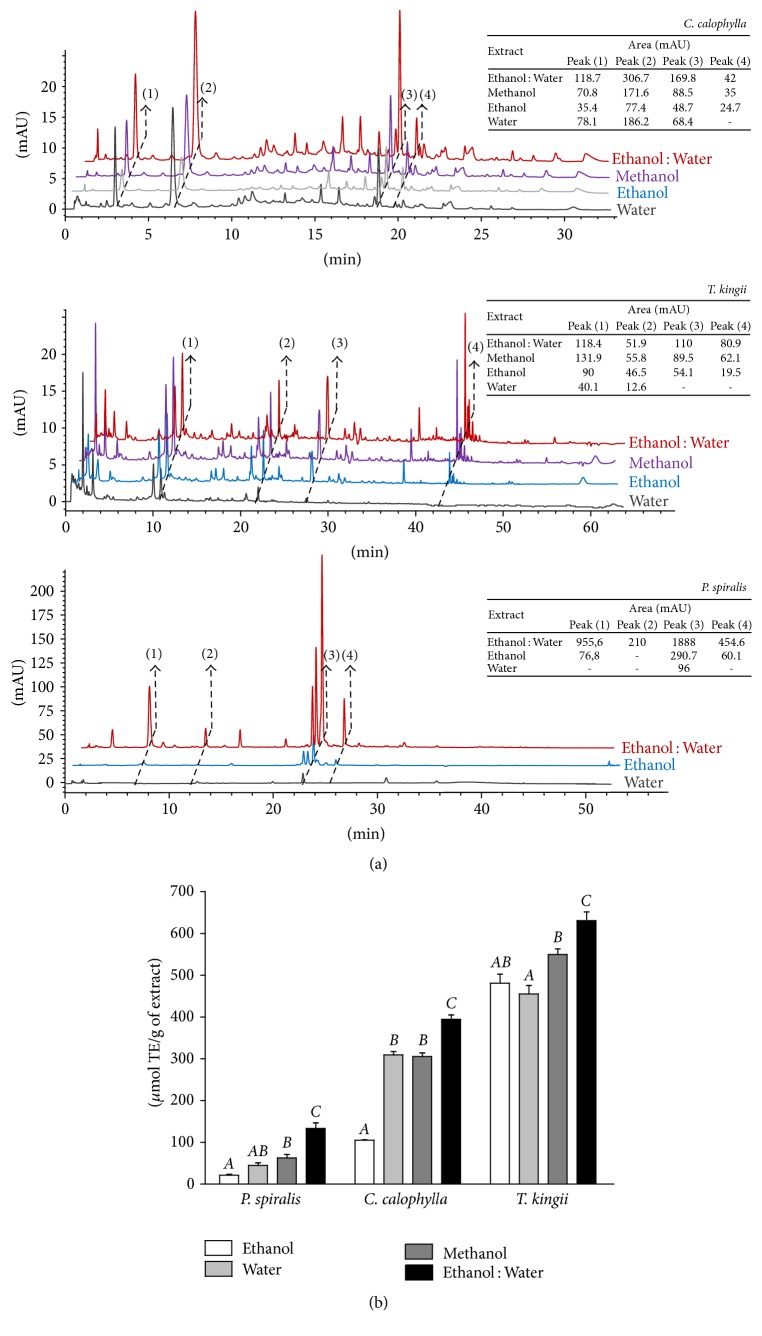
Ethanol-water extracts contain the best chromatographic fingerprint and antioxidant activity. Chromatographic fingerprint (a) and FRAP value (b) screening of ethanol, water, methanol, and ethanol-water (70 : 30 v/v) extracts prepared for* C. calophylla, T. kingii*, and* P. spiralis. *In the fingerprints, the areas of peaks are represented in terms of milli absorbance units (mAU). FRAP value (mean ± SD, *n* = 6) analysis was performed using one-way ANOVA; bars labeled with different letters indicate significant differences in each species (Bonferroni's multiple comparison test, *p* < 0.05).

In this study, the metabolite profile and antioxidant activity in four extracts at 100 *μ*g/mL were determined. Ethanol-water (70 : 30 v/v), methanol, ethanol, and water extracts were prepared from* C. calophylla, T. kingii*, and* P. spiralis. *To select extracts with the best qualities, the samples were evaluated by their chromatographic fingerprint using a HPLC-DAD technique, and antioxidant capacity was evaluated by FRAP (Figure 1). In the chromatographic analysis an Agilent Zorbax SB RRHT column was used to obtain separations with high resolution to improve the chromatographic information of the extract. Thus, for each species, the fingerprints of the extracts overlapped on the same chromatogram at 320 nm, and the areas of some randomly selected peaks were compared (Figure 1(a)). The highest chromatographic response in terms of resolution of peaks observed by DAD and areas of the selected peaks was obtained with the ethanol-water extract for the 3 species.

According to other studies, the chromatographic fingerprint is a characteristic profile which reflects the chemical composition of a sample and usually can be obtained using spectroscopic, electrophoretic, and chromatographic techniques [24]. However, due to their availability and versatility, the chromatographic techniques are widely used for the analysis of medicinal plants [25].

Regarding antioxidant capacity, the determined FRAP values showed the best activity in ethanol-water extracts (Figure 1(b)), which were significantly different compared with methanol, ethanol, and water extracts (*p* < 0.05). Therefore, the chromatographic and antioxidant information led to selection of the hydroalcoholic (ethanol-water) extracts of* C. calophylla *(HECC)*, T. kingii *(HETK), and* P. spiralis *(HEPS) because they presented the best properties according to this initial screening strategy.

### 3.2. Antioxidant Activity and TPC

Free radicals have been recognized to play a key role in the etiology of inflammation. They are mediators that provoke or sustain the inflammatory process; they are highly reactive, lead to oxidative stress, and trigger exacerbation of the inflammatory response [26]. Traditional medicinal plants usually have high antioxidant activity, and they are potential sources of bioactive compounds with therapeutic effects against diseases associated with the oxidative process [16, 17]. The antioxidant properties of HECC, HETK, and HEPS were determined using methodologies that consider different mechanisms of radical scavenging by hydrogen atom transfer and single electron transfer, such as the ORAC and FRAP assays, respectively [27]. These methods are frequently used to assess the potential of free radical stabilization in food and medicinal plant extracts [27, 28]. As shown in Table 1, the extracts showed FRAP values ranging from 835.310 to 4097.58 *μ*mol TE/g of extract. HETK exhibited the strongest reducing power (*p* < 0.05) compared to HECC and HEPS. The ORAC value of the extracts ranged from 3756.65 to 6494.26 *μ*g ET/g of extract (Table 1). HETK was again the best, since its result was significantly higher (*p* < 0.05) compared to the other species. It is well known that phenolic compounds constitute one of the main classes of natural antioxidants present in plants and exhibit anti-inflammatory effects in both* in vitro *and* in vivo* models [5, 29]. The determination of phenolic compounds in the extracts was performed using the Folin-Ciocalteu method, which is a simple and highly reproducible test for quantification of TPC. TPC ranged from 113.47 to 386.77 mg gallic acid equivalents (GAE)/g of extract. As shown in Table 1, the HETK extract showed the highest phenolic content (*p* < 0.05) when compared with HECC and HEPS, demonstrating that high phenolic content generally leads to high antioxidant activity.

### 3.3. Effects of Hydroalcoholic Extracts on the Viability of THP-1 Macrophages

**Figure 2:**
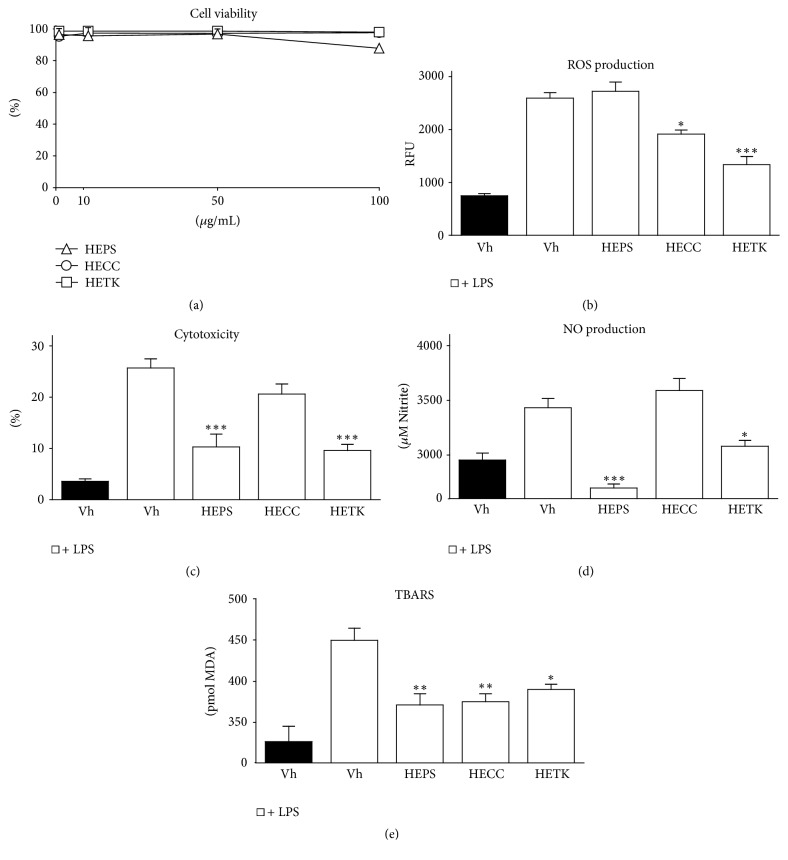
Effect of hydroalcoholic extracts on LPS-induced cytotoxicity and ROS/RNS production in THP-1 macrophages. Cell viability: cells were preincubated with the indicated concentrations of* P. spiralis *(HEPS),* C. calophylla *(HECC), and* T. kingii *(HETK) (a), ROS production (b), cytoprotective effect (c), NO production (d), and TBARS (e) were monitored in macrophages pretreated with 10 *μ*g/mL extract followed by 10 ng/mL LPS stimulation. Values are expressed as the mean ± SD (*n* = 3). Analysis was performed using one-way ANOVA; bars labeled with *∗* differ significantly compared with the control black bar (Dunnett's multiple comparison test, ^*∗*^*p* < 0.05, ^*∗∗*^*p* < 0.01, and ^*∗∗∗*^*p* < 0.001).

As shown in Figure 2(a), cytotoxicity was not observed when human THP-1 macrophages were exposed to HECC and HETK (1–100 *μ*g/mL) for 48 h, since cell viability was over 95%. In contrast, HEPS induced a significant diminution in the percentage of viability only at 100 *μ*g/mL; in the other concentrations (1–50 *μ*g/mL) cell viability was over 95% as with HECC and HETK. Therefore, extracts at 10 *μ*g/mL were selected to evaluate the anti-inflammatory activity and to exclude the possibility that effects were caused by cytotoxicity of the extracts on macrophage cells.

### 3.4. Hydroalcoholic Extracts of* C. calophylla* (HECC) and* T. kingii* (HETK) Inhibit LPS-Induced ROS Production in THP-1 Macrophages

ROS are highly reactive molecules with beneficial and detrimental effects. Moderate or low concentrations originating during the metabolic functions of the cell participate in signaling processes [26]. However, excessive production of ROS at sites of inflammation may result in hyperactivation of the inflammatory response and lead to the phenomenon of oxidative stress [30]. This event causes significant damage to biological systems such as lipids, proteins, and DNA and contributes to perpetuating the inflammatory process and the development of degenerative conditions [26]. Activation of macrophages by LPS induces the rapid production of ROS as one of the immediate defense mechanisms of the host. Therefore, it plays a key role in the progression of the excessive inflammatory response, and its modulation has been identified as a key objective in the assessment of the anti-inflammatory activity of natural products [31, 32]. To investigate the effect of HECC, HETK, and HEPS on LPS-induced ROS production, the levels of ROS were determined in THP-1 macrophages using the fluorescent probe DCFH-DA. As shown in Figure 2(b), the stimulation of macrophages with LPS showed a markedly increased level of ROS compared to the control group. LPS induced 3.4-fold overproduction of ROS compared with the basal levels, confirming the activation of the cells by this stimulus. Pretreatment with HECC and HETK significantly reduced the ROS levels 26.2% (*p* < 0.05) and 48.56% (*p* < 0.001), respectively, in LPS-induced macrophages. In contrast, HEPS extract did not reduce ROS production. The activity of HECC and HETK may be due to their high phenolic compound content and the antioxidant properties evidenced from the ORAC and FRAP assays (Table 1). HEPS did not show a regulatory effect on the production of ROS, probably due to its low phenolic compound content and less reducing power (Table 1). However, the pretreatment time could have been insufficient to have a significant effect.

### 3.5. Cytoprotective Effects of Hydroalcoholic Extracts in THP-1 Macrophages under LPS Stimulus

Upon stimulation of macrophages with LPS, signaling cascades are known to initiate the expression of inflammatory mediators including cytokines, interleukins, and increased ROS/RNS levels, which may induce deleterious effects [33, 34] and lead to the activation of apoptotic and necrotic pathways of cell death [35, 36]. To examine the impact of the hydroalcoholic extracts on LPS-induced cytotoxicity, LDH release was evaluated after LPS and extract cotreatment. As shown in Figure 2(c), the percentage of cytotoxicity demonstrates an increase (25.73%) of LDH release in LPS-stimulated macrophages. This was significantly attenuated by HEPS and HETK (*p* < 0.001). Pretreatment with HETK reduced cytotoxicity to 9.61%, whereas HEPS reduced cytotoxicity to 10.30%. In contrast, HECC did not present a significant cytoprotective effect on the cell death induced by LPS in macrophages. In a previous study, protective effects against LPS-induced oxidative stress were observed for other natural products such as tocopherols. Tocopherols and tocotrienols exhibit a high cytoprotective capacity against ROS and cytotoxicity induced by LPS [34]. In contrast, in the present study, HEPS did not decrease ROS production, but cytotoxicity was reduced. These results indicate that HEPS could regulate another pathway involved in the oxidative stress process with LPS stimulation. Upon stimulation these macrophages exhibit excessive accumulation of both NO and superoxide anion, whose interaction results in formation of toxic peroxynitrite (ONOO^−^) resulting in systemic inflammatory disorders. Thus, the inhibition of ROS production, iNOS, along with NO have been identified as therapeutic targets in screening of natural products [31].

### 3.6. Hydroalcoholic Extracts of* P. spiralis* (HEPS) and* T. kingii* (HETK) Inhibit LPS-Induced NO Production in THP-1 Macrophages

LPS can lead to the activation of a second level of inflammatory cascades, such as the expression of the iNOS isoenzyme in macrophages, inducing excessive production of NO in the cell [37]. NO is a molecule with important regulatory and effector functions that can act as a proinflammatory mediator under inflammatory conditions [35]. It plays a cytotoxic role under oxidative conditions because it may interact with the superoxide anion (O_2_^∙−^) to produce significant amounts of the most oxidatively active molecule, the peroxynitrite anion (ONOO^−^), which is a potent agent that can generate DNA fragmentation and lipid oxidation [26], contributing to the exacerbation of the inflammatory response. To determine the inhibitory properties of HECC, HETK, and HEPS on the LPS-induced production of NO, macrophages were incubated with these extracts and subsequently activated with LPS. The levels of NO in the culture medium were determined by using Griess reagent, which reacts with rapidly formed NO products as nitrate (NO_3_^−^) and nitrite (NO_2_^−^) anions [26]. As shown in Figure 2(d), stimulation with LPS markedly induced the production of NO compared to cells not stimulated with LPS. However, the pretreatment with HEPS and HETK significantly reduced NO production by 10.18% (*p* < 0.5) and 21.3% (*p* < 0.001), respectively. Additionally, no effect of HECC was detected on NO production in macrophages. These results provide evidence that* T. kingii *and* P. spiralis *could attenuate the inflammatory process through the downregulation of NO production. Due to the persistent production of proinflammatory mediators by macrophages and their close relationship with the onset of chronic and degenerative conditions, controlling the overproduction of these molecules is a potential strategy [38]. In fact, several in vitro and preclinical studies have demonstrated the anti-inflammatory properties of medicinal plants through the inhibition of NO production by the inhibition of iNOS [39].

### 3.7. Hydroalcoholic Extracts Suppress LPS-Induced TBARS Levels in THP-1 Macrophages

The inflammatory response is known to be accompanied by induction of oxidative and nitrosative stress pathways [18]. The overproduction of ROS/RNS can damage polyunsaturated fatty acids (PUFAs), one of the most important constituents of the phospholipids of cell membranes, in a process known as lipid peroxidation [40]. When the oxidative damage degree exceeds the capacity of repair, the mechanisms of cell death are activated and facilitate the development of pathological and degenerative states [41]. Lipid peroxidation originates a wide variety of secondary oxidation products, including MDA [41], a thiobarbituric acid reactive substance (TBARS) that is a highly mutagenic compound and classically used as a marker of oxidative/nitrosative stress [42]. Because HECC, HETK, and HEPS regulated ROS/RNS (Figures 2(b) and 2(d)), the effect of the extracts on the MDA lipid peroxidation marker was evaluated in macrophages stimulated with LPS (Figure 2(e)). Our results showed the ability of the extracts to decrease the quantified levels of MDA in cells. The pretreatment with HECC and HEPS caused significant reductions in MDA levels (*p* < 0.01) of 16.55% and 17.38%, respectively. While HETK significantly reduced the production of MDA (13.24% inhibition) compared with the vehicle control (*p* < 0.05), the reduction of MDA is an indicator of the final effect of* C. calophylla* and* T. kingii* on ROS production, as well as the ability of* P. spiralis* to interfere with NO production. In summary, the different inflammatory cell tests performed under LPS stimulus reveal the potential of these species to attenuate the inflammatory process in diverse ways.

### 3.8. Hydroalcoholic Extracts Attenuate LOX and COX in a Cell-Free System

**Figure 3:**
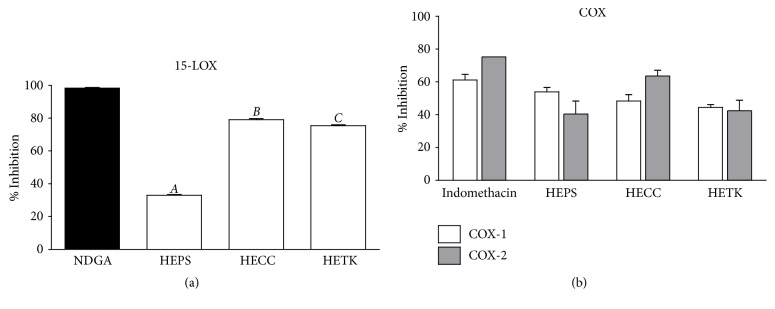
Suppression of 15-LOX and COX activity by the hydroalcoholic extracts of* P. spiralis* (HEPS),* C. calophylla* (HECC), and* T. kingii* (HETK). Percentage inhibition for 15-LOX (a) and COX (b) is expressed as the mean ± SD (*n* = 3). Analysis was performed using one-way ANOVA and *t*-test; bars labeled with different letters indicate significant differences between species at the same concentration (Bonferroni's multiple comparison test, *p* < 0.05).

PUFAs are found in large proportions in effector cells of the inflammatory response such as macrophages, neutrophils, and lymphocytes [40]. These lipids can be released from the cell membrane by the action of phospholipase enzymes in response to an activating stimulus derived from the inflammatory process [20]. Free PUFAs are substrates of enzymes from oxidative metabolism, including LOX and COX. The products from the action of LOX and COX are lipid mediators known as eicosanoids, which are involved in the intensity and duration of the inflammatory response [40, 43]. Specifically, high amounts of arachidonic acid (AA) have been reported as the main precursor of eicosanoids in inflammatory cells [40]. Since LOX and COX are involved in the biosynthesis of inflammatory mediators, the downregulation of their catalytic activity is an important target to avoid exacerbation of the inflammatory response. LOX is a family of enzymes that catalyze AA, the main substrate of this family of enzymes [44], into signaling compounds such as leukotrienes [45]. The LOX pathway is considered to be interesting in the treatment of a variety of inflammatory diseases [46]. The anti-inflammatory potential of HECC, HETK and HEPS was evaluated on 15-LOX, one of the most active isoforms of this enzyme predominantly expressed in immune effector cells [47]. NDGA at 52 *μ*M, a naturally occurring metabolite with potential anti-inflammatory and antioxidant activity [48], was used as a positive control. According to Figure 3(a), HEPS, HECC, and HETK exhibited inhibition of 15-LOX activity of 32.9%, 79.0%, and 75.4%, respectively.

COX enzymes are bifunctional and carry out one sequential reaction: the dioxygenation of AA and its respective reduction, in order to produce a series of final active compounds, among which are the prostaglandins (PGs) [43, 49]. These enzymes exist in two isoforms, COX-1 and COX-2, which have similar catalytic activity but are physiologically distinct [50]. COX-1 is a housekeeping enzyme, constitutively expressed throughout the body and of particular importance for gastrointestinal protection, vascular homeostasis, renal hemodynamics, and platelet function [51], whereas COX-2 is inducible in pathological conditions by inflammatory stimulation and plays a major part in the inflammatory process [43]. As a final product of the catalytic activity, the production of PGF_2*α*_ was quantified indirectly allowing the evaluation of the inhibitory activity of the extracts on these enzymes. Indomethacin, a nonselective inhibitor (5 *μ*M), was used as a positive control inhibitor. As a shown in Figure 3(b), HEPS was found to be most potent COX-1 inhibitor (53.9%), followed by HECC (48.4%) and HETK (44.4%). Regarding COX-2, HECC was decreased to 63.8% of the activity, followed by HETK (42.6%) and HEPS (40.7%). In general, the inhibition levels were greater than 40%, obtaining the highest inhibition for COX-2 (63.8%). In addition to the inflammatory properties of PGs generated by COX-1, they are also involved in several physiological functions; thus, COX-1 inhibition is linked to serious consequences such as gastric and intestinal ulcer formation [43, 51]. COX-2 has a different function playing an important role in the inflammatory responses of various tissues, so its inhibitors have been suggested to be potential anti-inflammatory due to their reduced or absent side-effects relative to those associated with inhibition of COX-1 [52].

Plant-derived secondary metabolites such as phenolic compounds have been reported to have the potential of inhibiting inflammatory reactions via suppression of the ROS/RNS, LOX, and COX pathways [34]. Generation of ROS/RNS, which act as secondary messengers and participate in signaling and cytotoxicity pathways [53], is strongly associated with acute and chronic inflammation [54]. In the same way, the eicosanoids also promote amplification of inflammatory signals and an influx in macrophages that, in turn, accelerate intracellular accumulation of ROS/RNS [3, 55]. Therefore, the ability of HECC, HETK, and HEPS to decrease ROS/RNS in LPS-stimulated THP-1 macrophages might be attributable to their ability to scavenge free radicals. This suggests that the HECC-, HETK-, and HEPS-mediated inhibition of ROS/RNS generation might also potentially inhibit the intracellular signaling cascade-dependent expression of proinflammatory mediators. In addition, HECC, HETK, and HEPS also showed inhibitory activity against COX and LOX, indicating anti-inflammatory properties that are ROS/RNS-independent. Hence, we speculate that the presence of phenolic compounds and antioxidant activity in HECC, HETK, and HEPS might be responsible for their anti-inflammatory activity. The overall results obtained in this study indicate for the first time the potential for these species to be used as anti-inflammatory agents [56], but they also provide a basis for directing research towards the identification of the phytochemical constituents responsible for their activity and thus contribute to the validation of their traditional use.

## 4. Conclusions

The results obtained demonstrate the active potential of* C. calophylla, T. kingii*, and* P. spiralis *for the modulation of key pathways in the development and perpetuation of the inflammatory process. Their ability to protect macrophages from toxic action mediated by LPS was demonstrated, which directly affects the inflammatory response that these cells can exert; their modulating activity on the production of radical species of oxygen and nitrogen was also demonstrated, which was complemented by demonstrating an ability to attenuate the process of lipid peroxidation, a fact that may be related to their antioxidant potential, since it was demonstrated that they are able to act through different mechanistic pathways. They also showed promising inhibitory activity on enzymes with high proinflammatory function. These results undoubtedly contribute to the fact that they are highly promising with the potential be considered for inclusion in the list of medicinal plants with accepted therapeutic purposes and the further development of active ingredients and/or standardized extracts.

## Figures and Tables

**Table 1:** TPC and antioxidant activity of hydroalcoholic extracts of *C. calophylla *(HECC)*, T. kingii *(HETK), and *P. spiralis *(HEPS).

Species	TPC (mg GAE/g)	FRAP (*µ*mol ET/g)	ORAC (*µ*mol ET/g)
HECC	180.51 ± 4.09^a^	1761.92 ± 3.05^a^	3756.65 ± 2.48^a^
HETK	386.77 ± 2.41^b^	4097.58 ± 2.89^b^	6494.26 ± 2.86^b^
HEPS	113.47 ± 3.26^c^	835.310 ± 2.50^c^	4216.56 ± 2.77^c^
Quercetin	Not applicable	11823.68 ± 1.02	24742.89 ± 4.16

mg GAE/g: milligrams gallic acid equivalents per gram of sample; *μ*mol ET/g: micromoles trolox equivalents per gram of sample. ^*∗*^Values (mean ± SD, *n* = 3) within each column followed by a different letter are significantly different (Bonferroni test, *p* value < 0.05).

## References

[1] Torres Carro R., Isla M. I., Ríos J. L., Giner R. M., Alberto M. R. (2015). Anti-inflammatory properties of hydroalcoholic extracts of Argentine Puna plants. *Food Research International*.

[2] Rajan T. S., Giacoppo S., Iori R. (2016). Anti-inflammatory and antioxidant effects of a combination of cannabidiol and moringin in LPS-stimulated macrophages. *Fitoterapia*.

[3] Fujiwara N., Kobayashi K. (2005). Macrophages in inflammation. *Current Drug Targets—Inflammation & Allergy*.

[4] Maitra U., Deng H., Glaros T. (2012). Molecular Mechanisms Responsible for the Selective and Low-Grade Induction of Proinflammatory Mediators in Murine Macrophages by Lipopolysaccharide. *The Journal of Immunology*.

[5] Iwalewa E. O., McGaw L. J., Naidoo V., Eloff J. N. (2007). Inflammation: the foundation of diseases and disorders. A review of phytomedicines of South African origin used to treat pain and inflammatory conditions. *African Journal of Biotechnology*.

[6] Jou I.-M., Lin C.-F., Tsai K.-J., Wei S.-J. (2013). Macrophage-mediated inflammatory disorders. *Mediators of Inflammation*.

[7] Ambriz-Pérez D. L., Leyva-López N., Gutierrez-Grijalva E. P., Heredia J. B., Yildiz F. (2016). Phenolic compounds: Natural alternative in inflammation treatment. A Review. *Cogent Food & Agriculture*.

[8] Shayganni E., Bahmani M., Asgary S., Rafieian-Kopaei M. (2016). Inflammaging and cardiovascular disease: Management by medicinal plants. *Phytomedicine*.

[9] Torres Carro R., D'Almeida R. E., Isla M. I., Alberto M. R. (2016). Antioxidant and anti-inflammatory activities of Frankenia triandra (J. Rémy) extracts. *South African Journal of Botany*.

[10] Hyun T. K., Kim H.-C., Kim J.-S. (2014). Antioxidant and antidiabetic activity of Thymus quinquecostatus Celak. *Industrial Crops and Products*.

[11] Wang Q., Kuang H., Su Y. (2013). Naturally derived anti-inflammatory compounds from Chinese medicinal plants. *Journal of Ethnopharmacology*.

[12] Dzoyem J. P., Eloff J. N. (2015). Anti-inflammatory, anticholinesterase and antioxidant activity of leaf extracts of twelve plants used traditionally to alleviate pain and inflammation in South Africa. *Journal of Ethnopharmacology*.

[13] Chanput W., Mes J. J., Wichers H. J. (2014). THP-1 cell line: An in vitro cell model for immune modulation approach. *International Immunopharmacology*.

[14] VÁSQUEZ R. (2016). SISTEMÁTICA DE LAS PLANTAS MEDICINALES DE USO FRECUENTE EN EL ÁREA DE IQUITOS. *Folia Amazónica*.

[15] Fonnegra-Gómez R., Villa-Londoño J. (2011). Plantas medicinales usadas en algunas veredas de municipios del altiplano del oriente antioqueño, Colombia. *Actual Biológicas*.

[16] Conforti F., Sosa S., Marrelli M. (2009). The protective ability of Mediterranean dietary plants against the oxidative damage: the role of radical oxygen species in inflammation and the polyphenol, flavonoid and sterol contents. *Food Chemistry*.

[17] Jiménez N., Carrillo-Hormaza L., Pujol A., Álzate F., Osorio E., Lara-Guzman O. (2015). Antioxidant capacity and phenolic content of commonly used anti-inflammatory medicinal plants in Colombia. *Industrial Crops and Products*.

[18] Maes M., Galecki P., Chang Y. S., Berk M. (2011). A review on the oxidative and nitrosative stress (O&NS) pathways in major depression and their possible contribution to the (neuro)degenerative processes in that illness. *Progress in Neuro-Psychopharmacology & Biological Psychiatry*.

[19] Wang L., Xu M. L., Liu J., Wang Y., Hu J. H., Wang M.-H. (2015). Sonchus asper extract inhibits LPS-induced oxidative stress and pro-inflammatory cytokine production in RAW264.7 macrophages. *Nutrition Research and Practice*.

[20] Dobrian A. D., Lieb D. C., Cole B. K., Taylor-Fishwick D. A., Chakrabarti S. K., Nadler J. L. (2011). Functional and pathological roles of the 12- and 15-lipoxygenases. *Progress in Lipid Research*.

[21] Fritsch-Decker S., Both T., Mülhopt S., Paur H.-R., Weiss C., Diabaté S. (2011). Regulation of the arachidonic acid mobilization in macrophages by combustion-derived particles. *Particle and Fibre Toxicology*.

[22] Ramana K. V., Reddy A. B. M., Tammali R., Srivastava S. K. (2007). Aldose reductase mediates endotoxin-induced production of nitric oxide and cytotoxicity in murine macrophages. *Free Radical Biology & Medicine*.

[23] Osorio E., Londoño J., Bastida J. (2013). Low-density lipoprotein (LDL)-antioxidant biflavonoids from Garcinia madruno. *Molecules*.

[24] Custers D., Van Praag N., Courselle P., Apers S., Deconinck E. (2017). Chromatographic fingerprinting as a strategy to identify regulated plants in illegal herbal supplements. *Talanta*.

[25] Goodarzi M., Russell P. J., Heyden Y. V. (2013). Similarity analyses of chromatographic herbal fingerprints: a review. *Analytica Chimica Acta*.

[26] Valko M., Leibfritz D., Moncol J., Cronin M. T. D., Mazur M., Telser J. (2007). Free radicals and antioxidants in normal physiological functions and human disease. *The International Journal of Biochemistry & Cell Biology*.

[27] Liang N., Kitts D. D. (2014). Antioxidant property of coffee components: Assessment of methods that define mechanism of action. *Molecules*.

[28] Huang D., Ou B., Prior R. L. (2005). The chemistry behind antioxidant capacity assays. *Journal of Agricultural and Food Chemistry*.

[29] Leopoldini M., Russo N., Toscano M. (2011). The molecular basis of working mechanism of natural polyphenolic antioxidants. *Food Chemistry*.

[30] Schieber M., Chandel N. S. (2014). ROS function in redox signaling and oxidative stress. *Current Biology*.

[31] Sekhar S., Sampath-Kumara K. K., Niranjana S. R., Prakash H. S. (2015). Attenuation of reactive oxygen/nitrogen species with suppression of inducible nitric oxide synthase expression in RAW 264.7 macrophages by bark extract of Buchanania lanzan. *Pharmacognosy Magazine*.

[32] Lara-Guzman O. J., Tabares-Guevara J. H., Leon-Varela Y. M. (2012). Proatherogenic macrophage activities are targeted by the flavonoid quercetin. *The Journal of Pharmacology and Experimental Therapeutics*.

[33] Kim I. D., Ha B. J. (2009). Paeoniflorin protects RAW 264.7 macrophages from LPS-induced cytotoxicity and genotoxicity. *Toxicology in Vitro*.

[34] Nishio K., Horie M., Akazawa Y. (2013). Attenuation of lipopolysaccharide (LPS)-induced cytotoxicity by tocopherols and tocotrienols. *Redox Biology*.

[35] Tripathi P., Tripathi P., Kashyap L., Singh V. (2007). The role of nitric oxide in inflammatory reactions. *FEMS Immunology & Medical Microbiology*.

[36] Redza-Dutordoir M., Averill-Bates D. A. (2016). Activation of apoptosis signalling pathways by reactive oxygen species. *Biochimica et Biophysica Acta (BBA) - Molecular Cell Research*.

[37] Cohen J. (2002). The immunopathogenesis of sepsis. *Nature*.

[38] Sautebin L. (2000). Prostaglandins and nitric oxide as molecular targets for anti- inflammatory therapy. *Fitoterapia*.

[39] Beg S., Swain S., Hasan H., Barkat M. A., Hussain M. S. (2011). Systematic review of herbals as potential anti-inflammatory agents: Recent advances, current clinical status and future perspectives. *Pharmacognosy Reviews*.

[40] Calder P. C. (2010). Omega-3 fatty acids and inflammatory processes. *Nutrients*.

[41] Ayala A., Muñoz M. F., Argüelles S. (2014). Lipid peroxidation: production, metabolism, and signaling mechanisms of malondialdehyde and 4-hydroxy-2-nonenal. *Oxidative Medicine and Cellular Longevity*.

[42] Tsikas D. (2017). Assessment of lipid peroxidation by measuring malondialdehyde (MDA) and relatives in biological samples: Analytical and biological challenges. *Analytical Biochemistry*.

[43] Martel-Pelletier J., Lajeunesse D., Reboul P., Pelletier J. (2003). Therapeutic role of dual inhibitors of 5-LOX and COX, selective and non-selective non-steroidal anti-inflammatory drugs. *Annals of the Rheumatic Diseases*.

[44] Haeggström J. Z., Funk C. D. (2011). Lipoxygenase and leukotriene pathways: biochemistry, biology, and roles in disease. *Chemical Reviews*.

[45] Joo Y.-C., Oh D.-K. (2012). Lipoxygenases: Potential starting biocatalysts for the synthesis of signaling compounds. *Biotechnology Advances*.

[46] Schneider I., Bucar F. (2005). Lipoxygenase inhibitors from natural plant sources, part 1: medicinal plants with inhibitory activity on arachidonate 5-lipoxygenase and 5-lipoxygenase/cyclooxygenase. *Phytotherapy Research*.

[47] Gulliksson M., Brunnström Å., Johannesson M. (2007). Expression of 15-lipoxygenase type-1 in human mast cells. *Biochimica et Biophysica Acta (BBA) - Molecular and Cell Biology of Lipids*.

[48] Lü J.-M., Nurko J., Weakley S. M. (2010). Molecular mechanisms and clinical applications of nordihydroguaiaretic acid (NDGA) and its derivatives: an update. *Medical Science Monitor*.

[49] Blobaum A. L., Marnett L. J. (2007). Structural and functional basis of cyclooxygenase inhibition. *Journal of Medicinal Chemistry*.

[50] Pasinetti G. M. (2001). Cyclooxygenase and Alzheimer's disease: Implications for preventive initiatives to slow the progression of clinical dementia. *Archives of Gerontology and Geriatrics*.

[51] Hawkey C. J. (2001). COX-1 and COX-2 inhibitors. *Best Practice & Research Clinical Gastroenterology*.

[52] Li W., Li H., Mu Q. (2014). Protective effect of sanguinarine on LPS-induced endotoxic shock in mice and its effect on LPS-induced COX-2 expression and COX-2 associated PGE 2 release from peritoneal macrophages. *International Immunopharmacology*.

[53] Brüne B., Dehne N., Grossmann N. (2013). Redox control of inflammation in macrophages. *Antioxidants & Redox Signaling*.

[54] Mittal M., Siddiqui M. R., Tran K., Reddy S. P., Malik A. B. (2014). Reactive oxygen species in inflammation and tissue injury. *Antioxidants & Redox Signaling*.

[55] Barbieri S. S., Eligini S., Brambilla M., Tremoli E., Colli S. (2003). Reactive oxygen species mediate cyclooxygenase-2 induction during monocyte to macrophage differentiation: critical role of NADPH oxidase. *Cardiovascular Research*.

[56] Carrillo-Hormaza L., Osorio E. (2017). Botanical ingredients: The key link in Colombia for the development of innovative and natural pharmaceutical, cosmetic, and food products. *Vitae*.

